# ROS-mediated genotoxicity of asbestos-cement in mammalian lung cells *in vitro*

**DOI:** 10.1186/1743-8977-2-9

**Published:** 2005-10-06

**Authors:** Elke Dopp, Santosh Yadav, Furquan Ahmad Ansari, Kunal Bhattacharya, Ursula von Recklinghausen, Ursula Rauen, Klaus Rödelsperger, Behnaz Shokouhi, Stefan Geh, Qamar Rahman

**Affiliations:** 1 Institute of Hygiene and Occupational Medicine, University Hospital Essen, Germany; 2 Fibre Toxicology Division, Industrial Toxicology Research Centre, Lucknow, India; 3 Institute of Physiological Chemistry, University Hospital Essen, Germany; 4 Institute of Occupational Medicine, University Hospital Giessen, Germany

**Keywords:** asbestos cement, chrysotile, cytotoxicity, micronuclei, kinetochore, free radicals

## Abstract

Asbestos is a known carcinogen and co-carcinogen. It is a persisting risk in our daily life due to its use in  building material as asbestos-cement powder. The present study done on V79-cells (Chinese hamster lung cells) demonstrates the cytotoxic and genotoxic potential of asbestos-cement powder (ACP) in comparison with chrysotile asbestos. A co-exposure of chrysotile and ACP was tested using the cell viability test and the micronucleus assay. The kinetochore analysis had been used to analyse the pathway causing such genotoxic effects. Thiobarbituric acid-reactive substances were determined as evidence for the production of reactive oxygen species. Both, asbestos cement as well as chrysotile formed micronuclei and induced loss of cell viability in a concentration- and time- dependent way. Results of TBARS analysis and iron chelator experiments showed induction of free radicals in ACP- and chrysotile exposed cultures. CaSO_4 _appeared to be a negligible entity in enhancing the toxic potential of ACP. The co-exposure of both, ACP and chrysotile, showed an additive effect in enhancing the toxicity. The overall study suggests that asbestos-cement is cytotoxic as well as genotoxic in vitro. In comparison to chrysotile the magnitude of the toxicity was less, but co-exposure increased the toxicity of both.

## Background

Asbestos has been well documented to be a carcinogen and co-carcinogen associated with the induction of mesothelioma, lung cancers and other benign lung diseases [1,2]. 'Asbestos' is a generic term for a group of six naturally occurring fibrous silicate minerals. It is grouped into two major classes: Serpentine, which contains a magnesium silicate called chrysotile and Amphibole, which includes crocidolite, amosite, anthophyllite, actinolite and tremolite [3]. Asbestos has been used in more than 3,000 products because of its high tensile strength, relative resistance to acid and temperature, varying textures and degrees of flexibility. It does not evaporate, dissolve, burn, or undergo significant reactions with other chemicals, which make asbestos non-biodegradable and environmentally cumulative. Over 95% of the total commercial asbestos use all over the world is chrysotile asbestos [4]. Chrysotile has the morphology of being curly and pliable [5]. Size, geometry, chemical composition and surface charge of various asbestos types play an important role in interactions with cells that lead to cell injury and disease [6,7]. Respiratory impairment, bronchial asthma, chronic bronchitis was noticed in asbestos cement factory workers [8]. However, in the case of chrysotile asbestos, its positive surface charge is more important than its morphology in rendering a toxic and lytic potential [9]. The iron content in chrysotile, primarily present as a surface contaminant [7] is low (~1–6%), but has to be considered in its toxicity.

Asbestos fibres in the environment can result from mining, milling and weathering of asbestos-bearing rocks, and from the manufacture, wear, and disposal of asbestos-containing products [10]. Because of the widespread use of asbestos, its fibres are ubiquitous in the environment. Indoor air can become contaminated with fibres released from building materials, especially if they are damaged or crumbling. Common sources of asbestos in homes are ceilings, pipe insulation, boiler coverings, wallboard, floor, ceiling tiles, sheets, pipes and jointings, etc. Asbestos-cement products, e.g. roof tiles, contain as much as 11–12% of chrysotile asbestos. As a result of continuing exposure to the weather and to acid rain, the surface of asbestos-cement products becomes corroded and weathered. Cement particles, asbestos fibres and agglomerates of particles and fibres are therefore released from the surface and may be dispersed in air and water in large amounts [11].

The toxicity of asbestos is characterized by a number of processes, among which the production of reactive oxygen and reactive nitrogen species (ROS and RNS) are thought to be the most important ones. Highly reactive oxygen species such as the hydroxyl radical can be produced through Fenton-type reaction catalysed by iron impurities present on the surface. ROS/RNS are also produced in the lungs by the chronic inflammatory reaction produced by the prolonged phagocytic activity of macrophages against the bio-persistent fibres [12]. ROS/RNS can cause various types of DNA damages. The most extensively studied are lesion of 8-oxodeoxyguanosine (8-oxodGuo) or the corresponding base (8-oxoGua). These altered nucleotides can be detected in the DNA of cell lines of human or animal origin after treatment with asbestos fibres [13,14].

Smailyte *et al*. [15] analyzed the cancer risk in Lithuanian cement producing workers and found that exposure to cement dust may increase lung and bladder cancer. He further reported a dose related risk for stomach cancer. Fatima *et al*. [16] have reported chromosomal abnormalities in asbestos cement factory workers. Rahman *et al*. [17] found chromosomal aberrations, sister chromatid exchanges and micronuclei formation in the blood lymphocytes of asbestos cement factory workers in comparison to their controls. Dušinská *et al*. [12] investigated chromosomal and DNA damage in former asbestos cement plant workers. As discussed above chrysotile is the most commercially exploited variety of asbestos and mostly used as asbestos-cement for building material. There are not many studies that assess the cyto- and genotoxicity of asbestos cement in vitro using cell lines. *In the present study*, we have investigated if asbestos-cement causes similar effects in cellular systems regarding cytotoxicity and genotoxicity than chrysotile asbestos. The micronucleus assay was applied to test the genotoxic effects of asbestos cement in V79-cells (Chinese hamster lung cells), an established cell culture model. Application of kinetochore analysis, radical measurements and iron chelator experiments gave more informations about the mechanistic background, which seems to be based on the formation of free radicals.

## Results

Light microscopy showed the average percentage of fibre sizes in asbestos-cement samples to be 50.3% (< 5 – 10 μm), 31.2% (11–20 μm) and 18.5% (21 – 30 μm) and in chrysotile 49.7% (< 5 – 10 μm), 30.7% (11 – 20 μm) and 19.5% (21 – 30 μm) (Table 1). The cytotoxic potential of asbestos cement, chrysotile asbestos and CaSO_4 _(negative control) was determined after an exposure time of 24, 48 and 72 hrs. The results show a decrease in cell viability of ACP- and chrysotile-exposed V79-cells with increasing fibre/dust concentrations and exposure times. The results showed chrysotile to be more cytotoxic than the ACP after 24, 48 and 72 hrs exposure (Figure 2). CaSO_4 _was seen to be negligibly cytotoxic up to the highest concentration (20 μg/cm^2^) and also did not have any effect at longer exposure times.

**Table 1:** Analysis of asbestos-cement and chrysotile samples using light microscopy (magnification: 2000×) Data represent the mean of 33 counting.

**Sample**	**WHO-fibres F/ml × 10**^6^	**% of fibres/sample**	**Distribution of fibres according to the length (%)**		
			
			**< 5–10 μm**	**10–12 μm**	**20–30 μm**
Asbestos cement	144	12.8	50.3	31.2	18.5
Chrysotile	697	100	49.7	30.7	19.5

**Table 2:** Fibre counting by electron microscopy

**Sample**	**Suspension**			**WHO-fibre counts**			**TEM**
	mg/ml		Filter deposit		F/mg	F/ml	Magnification, Number of fields
	Original	Diluted	μg/cm^2^	n	×10^6^	×10^6^	
Asbestos cement	11	0.05	73.2	14	**38.3**	**425**	×10000, 10 fields
				34	**12.9**	**144**	×2000, 2 fields
UICC-chrysotile	5	0.01	13.2	32	**486**	**2432**	×10000, 10 fields
				33	**139**	**697**	×2000, 1 field

**Figure 1:**
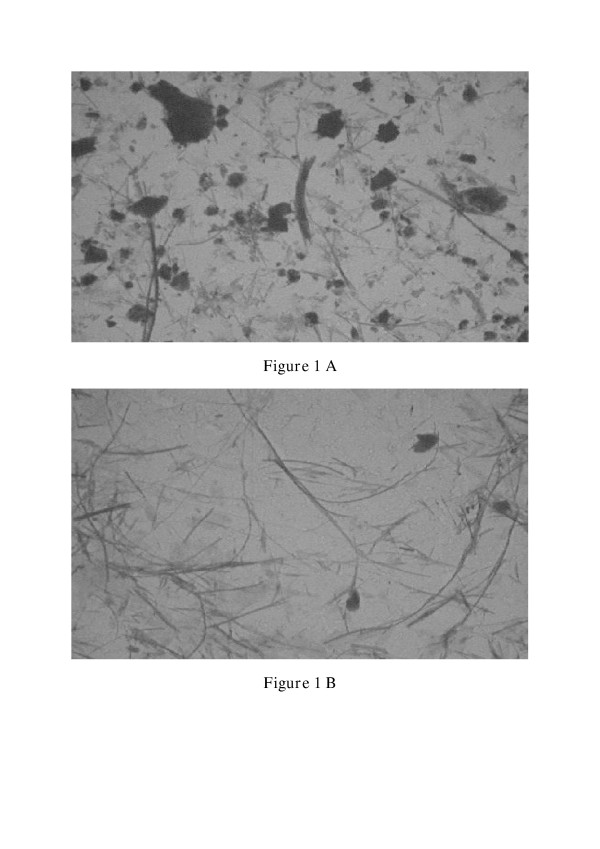
Transmission electron microscopy pictures of asbestos-cement **(A) **and chrysotile **(B) **samples. (Magnification: 2000×)

**Figure 2:**
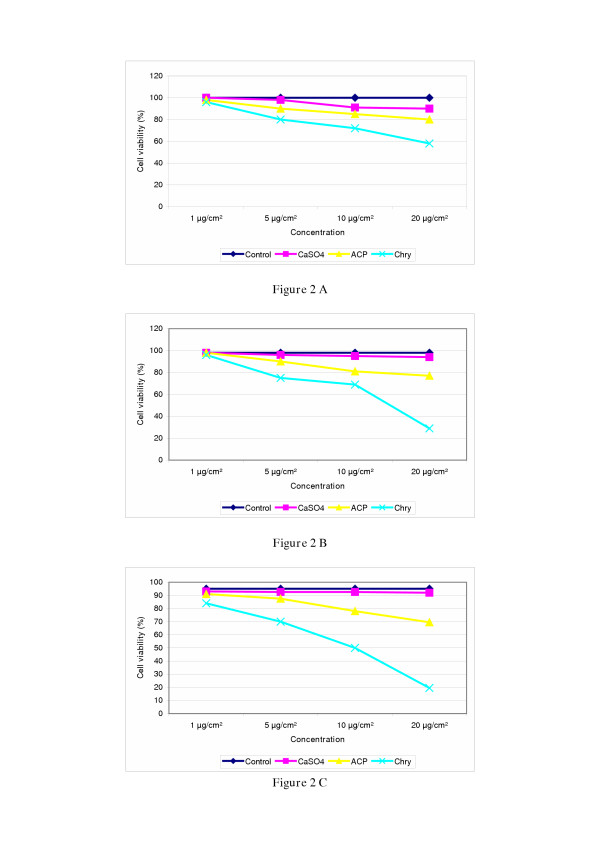
Cytotoxicity of asbestos-cement in V79-cells. Cells were treated with various doses (1 μg/cm^2^, 5 μg/cm^2^, 10 μg/cm^2^, 20 μg/cm^2^) of asbestos-cement and chrysotile for **24 hrs (A), 48 hrs (B) and 72 hrs (C)**. The percentage of decreased cell viability is shown in relation to the untreated control. The cytotoxicity was determined by trypan blue-staining. The experiments were repeated twice. SD < 1%

**Figure 3:**
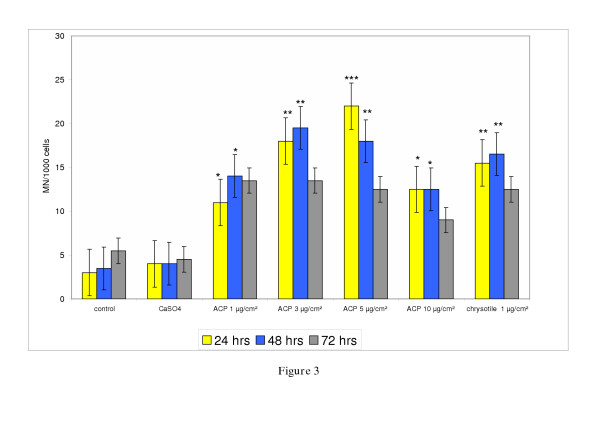
Micronucleus induction after exposure of V79-cells to different doses of asbestos-cement for 24, 48 and 72 hrs. The experiment was repeated twice. Significance: * p < 0.05, ** p < 0.01, *** p < 0.001.

**Figure 4:**
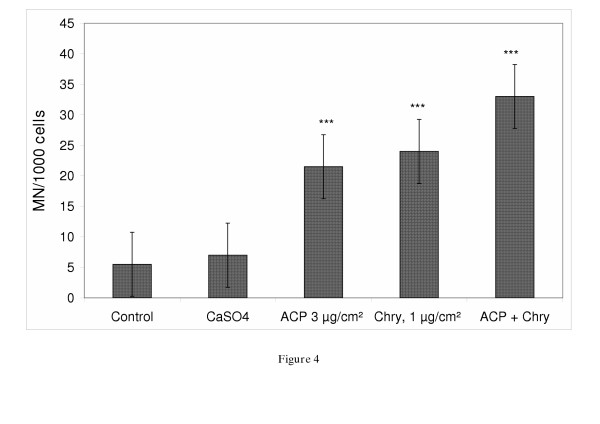
Micronucleus induction in V79-cells after co-exposure of asbestos and asbestos- cement (chrysotile: 1 μg/cm^2^, ACP: 3 μg/cm^2^; exposure time: 48 h). The experiment was repeated twice. Significance: *** p < 0.001.

Figure 3 shows the level of induced micronuclei (MN) by ACP in V79-cells at 24, 48 and 72 hrs consecutively to a concentration of 1, 3, 5 and 10 μg/cm^2 ^of ACP. ACP induced a significant number of micronucleated cells at all applied concentrations after 24 hrs and 48 hrs of exposure with the highest induction at 5 μg/cm^2 ^after 24 hrs. The reduced number of MN after 72 hrs exposure can be explained by increased cytotoxic effects at the applied ACP-concentrations. On comparing ACP to chrysotile the latter induced at a concentration of 1 μg/cm^2 ^almost equal numbers of MN as ACP at a concentration of 3 μg/cm^2 ^(p < 0.01). The results of a co-exposure of ACP (3 μg/cm^2^) and chrysotile (1 μg/cm^2^) are shown in Figure 4. Additive effects can be seen through an increased formation of MN as compared to induction by ACP (3 μg/cm^2^) or chrysotile (1 μg/cm^2^) alone. The difference between ACP or chrysotile alone and co-exposed V79-cells is statistically not significant.

The kinetochore analysis revealed a slight increase in kinetochore-negative micronuclei in cells exposed to ACP (3 μg/cm^2^), chrysotile (3 μg/cm^2^) and co-exposure of ACP (3 μg/cm^2^) and chrysotile (1 μg/cm^2^) indicating clastogenic events (p < 0.05). However, the differences compared to the untreated control are statistically not significant. CaSO_4 _induced a negligible amount of kinetochore-negative micronuclei, which was almost equal to controls. Co-exposure of ACP and chrysotile induced kinetochore-negative micronuclei almost equal to that induced by chrysotile (1 μg/cm^2^) alone (Table 3).

**Table 3:** Kinetochore analysis after exposure of V79-cells to asbestos cement, chrysotile and gypsum (negative control) for 48 h. The experiment was repeated twice.

**Samples**	**No of scored MN**	**Mean K^- ^(± SD)**
Control	200	68.5 ± 0.70
CaSO_4 _(3 μg/cm^2^)	200	67 ± 2.12
Asbestos cement (3 μg/cm^2^)	200	71.5 ± 1.4
Chrysotile (1 μg/cm^2^)	200	73.5 ± 0.70
Co-exposure (3 μg/cm^2 ^ACP and 1 μg/cm^2 ^chrysotile)	200	75 ± 2.8

**Figure 5:**
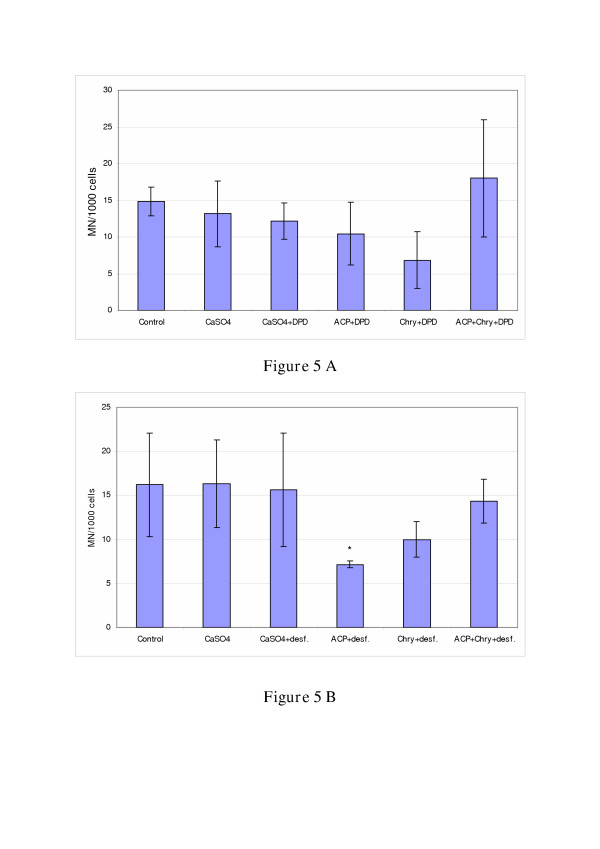
Reduction of micronucleus formation in exposed V79-cells after addition of the iron chelators 2,2'-dipyridyl (final concentration: 100 μM) **(A) **and desferal (final concentration: 100 μM) **(B)**. The treatment of cells with 2,2'-DPD and desferal, respectively, was done simultaneously with the fiber and particle treatment (concentrations: 3 μg/cm^2 ^CaSO_4_; 3 μg/cm^2 ^asbestos cement; 1 μg/cm^2 ^chrysotile; exposure time: 48 h).

Addition of the iron chelators 2,2'-DPD and desferal reduced the number of induced micronuclei (see Fig. 4) to the control level or even lower (Figure 5). The iron chelators 2, 2' DPD and desferal are able to prevent radical formation in cellular systems by complexation of free metal and iron ions. A stronger reducing effect in MN-formation can be observed in chrysotile-exposed V79 cells compared to asbestos cement-exposed cells (Fig. 5A). However, application of desferal induced stronger reducing effects in ACP-exposed V79-cells (Fig. 5B). This reduction is statistically significant (p < 0.05).

**Figure 6:**
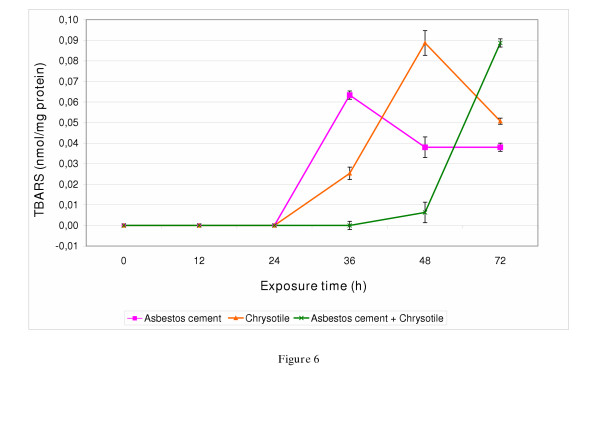
Thiobarbituric acid-reactive substances (TBARS) released by V79-cells exposed to asbestos-cement (3 μg/cm^2^), chrysotile (1 μg/cm^2^) or asbestos cement and chrysotile in co-exposure. The experiment was repeated twice. Significance: * p < 0.05

The formation of TBARS was detected in ACP, chrysotile and co-exposed (ACP and chrysotile) V79-cells (Figure 6). Fe/8HQ (1.6 μl/ml) was used as positive control and induced TBARS formation up to 0.25 nmol/mg protein. After 24 h exposure to ACP or chrysotile, V79-cells started to release low levels of TBARS, which enhanced in quantity longer exposure times of 36 h (0.063 nmol/mg, ACP) and 48 h (0.089 nmol/mg, chrysotile), respectively. We observed a delayed formation of TBARS after co-exposure of V79-cells to ACP and chrysotile after 72 h exposure time (0.089 nmol/mg) (Figure 6).

## Discussion

The present study demonstrates a time- and concentration- dependent loss of cell viability in chrysotile-exposed V79-cells. These results are in agreement to those found by Hong and Choi [18] in V79-cells. The genotoxicity analysis using micronuclei (MN) as biomarker proved that chrysotile gave a maximum damage to the cells at relatively low concentrations. Similar observations were also found in our earlier studies done with human mesothelial cells (HMC) [19-21] and human peripheral blood lymphocytes [22]. The studies suggest that clastogenic factors are responsible for the genotoxic effect shown as kinetochore-negative MN. In the past Dopp *et al*. [23], Dopp and Schiffmann [24], Rahman *et al*. [3] and Poser *et al*. [21] have shown that clastogenic events caused by chrysotile are responsible for the formation of micronuclei in different cell types. The results of the TBARS analysis in the present study further strengthened chrysotile-induced clastogenic events by suggesting the release of free radicals. The present study showed AC-induced release of TBARS (evidence for lipid peroxidation) after 24 – 48 hrs exposure. The highest amount of MN induction in V79-cells was also found during this period demonstrating an interrelation between these two features. In the case of chrysotile asbestos, a delayed release of TBARS (> 24 hrs exposure) can also be observed. These findings are in concordance with findings of Burmeister *et al*. [25]. These authors did not observe an increase in Fpg-sensetive sites indicative of oxidative DNA-base modification in asbestos-treated human mesothelial cells up to an exposure time of 24 hrs. Abidi *et al*. [26] and Afaq *et al*. [27] reported about the production of high amounts of TBARS and alteration of the GSH redox system by chrysotile fibres. Kopnin *et al*. [28] showed that fibroblasts as well as mesothelial cells are able to generate reactive oxygen species (ROS) in response to asbestos exposure whereas fibroblasts have a lower ability to produce ROS compared to mesothelial cells.

ACP induced pronounced cytotoxicity and genotoxicity in V79-cells, even though its toxic effects were lower than that of chrysotile both in dosage and induction levels. Tilkes and Beck [29] reported similar findings on macrophages in which asbestos cement caused lower cytotoxicity than UICC-chrysotile. Exposure to the different concentrations of ACP showed increased formation of micronuclei in V79-cells.

The co-exposure of V79-cells to ACP and chrysotile resulted in a weak additive effect. However, the amount of induced kinetochore-negative MN did not vary much from that induced by chrysotile. **In summary**, it can be stated that both ACP and chrysotile have cytotoxic and genotoxic properties. However, the toxicity of chrysotile is more pronounced than that of ACP. The co-exposure (ACP and chrysotile) of V79-cells showed weak additive genotoxic effects. The release of TBARS in ACP- and chrysotile exposed V79-cells suggests the involvement of free radicals in fibre/dust-induced toxicity.

## Methods

### Fibres and dust samples

Asbestos cement powder (ACP) was prepared from asbestos cement sheet by grinding with mortar and pestle (Industrial Toxicology Research Centre, Lucknow, India). The main type of asbestos fibre in the ACP-sample is chrysotile asbestos (12.8% fibres/sample, Tab. 1). The sample was sieved through a 30 μm brass sieve. Sterilization was carried out at 120°C for 2 hours (hrs) and the samples were subsequently suspended in sterile PBS buffer. The suspensions were analysed by transmission electron microscopy (Hitachi H600) according to fibrous and non-fibrous material and the number of fibres/ml were calculated according to the counting rules of VDI 3492 (VDI Richtlinie, 1994) (Tab. 2). Further fibre and particle analyses were carried out using light microscopy [30] and transmission electron microscopy (TEM, Phillips). TEM pictures of asbestos-cement and chrysotile samples are shown in Fig. 1 (magnification: 2000×). The large non-fibrous conglomerate material in Fig. 1A represents agglomerates of cement particles.

A final concentration of asbestos cement powder (ACP) in PBS (phosphate buffered saline) of 1.1 mg/ml was used. Chrysotile (UICC) and commercially available CaSO_4 _(Sigma, Taufkirchen, Germany) were applied as positive and negative controls, respectively.

### Cell culture

V79-cells (Chinese hamster lung fibroblast cells) were obtained from ECC (European Collection of Cell Cultures, ECC No.: 86041102). Cells were cultivated in RPMI-1640 (Gibco) with Fetal Calf Serum (10%) (Gibco), L-glutamine (1%) (Gibco) and antibiotics (100 U/ml penicillin and 100 μg/ml streptomycin) (Gibco) at 37°C and 5% CO_2_.

### Cytotoxicity test

V79-cells (state of confluence: max. 70%) were treated with ACP, CaSO_4 _and chrysotile, respectively, at different doses (1 μg/cm^2 ^– 10 μg/cm^2^) for 24 hrs, 48 hrs and 72 hrs. Cell viability was evaluated immediately after exposure. Treated and untreated cells were harvested by trypsin treatment (Sigma). Cell counting was performed following trypan blue staining. The cell suspension was mixed with an equivalent volume of 0.4% trypan blue solution (Sigma) and subsequently evaluated under the light microscope. The membrane of dead cells is permeable to trypan blue (blue stained cells), whereas living cells remain unstained. Cell viability is expressed as percentage of surviving cells compared to the total number of cells. A substance is considered to be cytotoxic if the decrease in cell viability is ≤ 50%.

### Micronucleus assay and kinetochore analysis

For micronucleus (MN) analysis, 2 × 10^5 ^V79-cells were seeded in each well of Quadriperm-dishes (Viva-Science, Sartorius, Göttingen, Germany) and cultured overnight. Then the fibre and dust samples were applied for 24 hrs, 48 hrs or 72 hrs at different concentrations. At the end of the exposure times cells were fixed and stored in cold methanol (-20°C) for at least 30 minutes before staining. For micronucleus assay the cells were washed with PBS/CMF (calcium- and magnesium-free phosphate buffered saline) and the nuclei were stained with bisbenzimide (Hoechst 33258, concentration: 5 μg/ml, 4 minutes). The slides were then mounted for fluorescence microscopy and examined for the presence of micronuclei. Each data point represents the mean of 3 treated cultures from different experiments with 2000 nuclei evaluated in each case. The significance was tested by using the Chi^2^-test.

For further analysis of the induced micronuclei after treatment of cells with ACP or chrysotile, kinetochores were stained by incubating the fixed cell preparations with CREST antibodies (Chemicon, Temecula, CA, U.S.A.) for 1 hr in a humidified chamber at 37°C. After rinsing with PBS with 0.5% Tween 20 (Sigma, Germany), the cells were incubated with fluorescein isothiocyanate (FITC)-conjugated anti-human IgG (Antibodies Incorporated, Davis, USA) for 30 min before applying bisbenzimide. At least 200 micronuclei were examined for the presence of kinetochores in each case. The significance was tested by using the Chi^2^-test.

### Application of iron chelators

The iron chelators 2,2'-dipyridyl (DPD) (Fluka, Germany) and desferal (Novartis, Germany) were used to investigate the reduction of the particle induced genomic effects by binding to metal/iron ions. Herewith, the formation of free radicals can be reduced. DPD (final concentration: 100 μM) and desferal (final concentration: 10 mM) were dissolved in ddH_2_O and added to the culture medium. The treatment of cells with DPD or desferal was done simultaneously with the fibre/dust treatment (concentrations: ACP 3 μg/cm^2^, chrysotile 1 μg/cm^2^, CaSO_4 _as negative control 3 μg/cm^2^, exposure time: 48 hrs).

### Radical measurement

Thiobarbituric acid-reactive substances (TBARS) were determined as indication for the formation of reactive oxygen species. TBARS were determined in the supernatant after various incubation times. V79-cells were cultivated in Ham's F12 culture medium for 24 hrs. The cells were then exposed to asbestos cement (3 μg/cm^2^), chrysotile (1 μg/cm^2^) or asbestos cement and chrysotile in co-exposure for 6 h, 12 h, 24 h, 36 h, 48 h, and 72 h. Cells cultured in Ham's F12 medium were used as negative control. After the different exposure times, 1 ml of the probe was mixed with 200 μl iced trichloracetic acid (30%) to precipitate the protein and thereafter centrifuged at 10,000 rpm for 5 min. Further, 1 ml of the supernatant was incubated with 500 μl thiobarbituric acid (1%) in a water bath at 95°C for 10 min. After centrifuging at 3000 rpm for 5 min, the absorbance of the supernatant was measured in a spectral photometer at 532 nm. The amount of TBARS formed was expressed as malondialdehyde (MDA) equivalents in the supernatant. The concentration of the TBARS was calculated by a calibration curve (standard substance: 1, 1, 3, 3-tetramethoxypropane, Sigma, Germany). The experiments were performed in duplicates.

### Statistical analysis

The chi^2^-test was used for comparison of micronucleus and kinetochore results with the untreated control in each set of experiments.

## Competing interests

The author(s) declare that they have no competing interests.

## Authors' contributions

QR had the initial idea of performing the investigations together with ED, who had coordinated the experiment and had edited the final version of the manuscript. SY and SG have carried out the genotoxicity studies. KB had drafted the manuscript and BS had participated with him in the genotoxicity studies with different iron chelators. UvR has done the cytotoxicity studies and the TBARS assay with help of UR. FAA had done the light microscopic study of the samples. Finally, KR had done the electron microscopic study of the samples. All authors read and approved the final manuscript.
